# Citrate lyase CitE in *Mycobacterium tuberculosis* contributes to mycobacterial survival under hypoxic conditions

**DOI:** 10.1371/journal.pone.0230786

**Published:** 2020-04-17

**Authors:** Jialing Hu, Kaixi Jin, Zheng-Guo He, Hua Zhang

**Affiliations:** College of Life Science and Technology, National Key Laboratory of Agricultural Microbiology, Huazhong Agricultural University, Wuhan, China; Institut de Pharmacologie et de Biologie Structurale, FRANCE

## Abstract

*Mycobacterium tuberculosis* is the causative agent of tuberculosis and has evolved an ability to survive in hostile host environments. *M*. *tuberculosis* is thought to utilize the rTCA cycle to sustain its latent growth during infection, but the enzymatic characteristics and physiological function for the key citrate lyase of the rTCA cycle, MtbCitE, in the important pathogen remain unclear. In this study, we investigated the function of MtbCitE based on its structural properties and sequence comparisons with other bacterial citrate lyase subunits. We showed that several amino acid residues were important for the citrate cleavage activity of MtbCitE. Strikingly, the citrate cleavage activity of MtbCitE was inhibited by ATP, indicating that energy metabolism might couple with the regulation of MtbCitE activity, which differed from other CitEs. More interestingly, deletion of *citE* from *Mycobacterium bovis* BCG decreased the mycobacterial survival rate under hypoxic conditions, whereas complementation with *citE* restored the phenotype to wild-type levels. Consistently, three key rTCA cycle enzymes were positively regulated under hypoxic conditions in mycobacteria. Therefore, we characterized a unique citrate lyase MtbCitE from *M*. *tuberculosis* and found that the CitE protein significantly contributed to mycobacterial survival under hypoxic conditions.

## Introduction

*Mycobacterium tuberculosis* infects one-third of the total human population because it can survive within the host for a long time in a latent form. Hypoxia is thought to induce a state of non-replicating persistence within *M*. *tuberculosis* and play a significant role in its growth, but the underlying mechanism remains unclear [1, 2]. Previous studies demonstrated that *M*. *tuberculosis* may switch its metabolism pathway to a less energy-efficient status to adapt to oxygen-limiting conditions, leading to low ATP concentration in hypoxic cells [3]. The reductive side of the tricarboxylic acid (TCA) cycle is an important part of this strategy, and is shared by most of the enzymes in the TCA cycle; moreover, it allows carbon fixation under anaerobic conditions [4, 5]. Citrate lyase is one of the key enzymes of the rTCA cycle [6].

Citrate lyase is a cytoplasmic enzyme that catalyzes the conversion of citrate and CoA into oxaloacetate and acetyl-CoA. Acetyl-CoA is an important molecule in cellular metabolism, that is, used in the biosynthesis of a diverse set of molecules (e.g., fatty acids and cholesterol), energy production, and protein acetylation [7] As such, citrate lyase is widely distributed among eukaryotes, archaea, and bacteria. In eukaryotes, citrate is metabolized into acetyl-CoA and oxaloacetate by ATP-citrate lyase, which is composed of single polypeptide chains [8]. However, in most bacteria, citrate lyase consists of a complex comprising three non-identical subunits: enzymatic α and β subunits, encoded by *citD* and *citE*, respectively, and acyl-carrier γ subunit, encoded by *citF* [9]. The α subunit functions as an acetyl-ACP transferase, the β subunit is a citryl-S-ACP lyase, and the γ subunit is an acyl carrier protein [10, 11]. In bacteria, the cleavage of citrate by the citrate lyase complex depends on Mg^2+^, but ATP, which is important for citrate cleavage in eukaryotes, has no effect on the activity of citrate lyase [12, 13].

In pathogenic bacteria, such as *M*. *tuberculosis*, citrate lyase lacks the α and γ subunits, and is directly annotated as CitE [14]. Thus, the biochemical function of citrate lyase in *M*. *tuberculosis* may differ from that in bacteria containing the complete citrate lyase complex. According to Arora G. et al, there are two homologous genes (Rv2498c and Rv3075c) in *M*. *tuberculosis* [15], but in this study, we only focus on the previous accepted CitE (Rv2498c). The structure of *M*. *tuberculosis* CitE (MtbCitE) was resolved by Goulding et al., who suggested that several amino acid residues play a significant role in the catalytic function of MtbCitE [14]. The complex structures of MtbCitE with oxaloacetate and magnesium were also resolved, and several amino acid residues were found to be involved in the binding of Mg^2+^ and oxaloacetate. Interestingly, ATP molecules were also proposed to be bound by MtbCitE; however, no direct evidence of this binding was obtained.

In this work, we investigated the function of MtbCitE based on its structural properties and sequence comparisons with other bacterial citrate lyase subunits. We found that MtbCitE demonstrated citrate lyase activity by itself, and the function of MtbCitE was inhibited by ATP. Furthermore, deletion of *citE* from *Mycobacterium bovis* BCG has been proven to be associated with the decreasing survival rate of mycobacteria under hypoxic conditions. Therefore, we sought to expand our understanding of the surviving mechanism of *M*. *tuberculosis*.

## Materials and methods

### Bacterial strains, plasmids, enzymes, and chemicals

*E*. *coli* BL21 (DE3) and pET28a containing the T7 RNA polymerase promoter were purchased from Novagen and used to express CitE protein and its mutants. Restriction enzymes, T4 ligase, dNTPs and all antibiotics were purchased from TaKaRa Biotech, whereas DNA purification kits were purchased from Watson Biotechnologies. Ni-NTA agarose columns were obtained from Qiagen.

### Bacterial culture and hypoxic assay

The *M*. *bovis* BCG Pasteur 1173P2 and *M*. *tuberculosis* H37Ra strains were pre-cultured in Middlebrook 7H9 supplemented with 0.2% glycerol, 10% OADC and 0.05% Tween 80 (v/v). The bacterial suspension was adjusted to an optical density at 600 nm (OD_600_) of 1.0 and then cultured at 37 °C [16]. To investigate the gaseous conditions on bacterial survival, bacteria were cultured under hypoxic or normoxic conditions. Three milliliters of culture was injected into 5-mL uncoated vacutainer tubes and incubated in a static position at 37 °C [17]. Control cultures containing methylene blue (1.5 μg/mL) were used to monitor the depletion of oxygen. Cells were harvested after 72 hours of hypoxia culture and processed for survival measurement and RNA isolation [17]. The survival of BCG strains was determined by CFU assay after 14 days culturing on 7H10 plates.

### Quantitative real-time PCR analysis

The expressions levels of citrate lyase and 2-oxoglutarate synthase of *M*. *tuberculosis* H37Ra and *M*. *bovis* BCG Pasteur 1173P2 in different conditions were investigated by Quantitative real-time PCR analysis. Isolation of mRNA and cDNA from mycobacterial strains was performed as described previously [18]. For real-time PCR analysis, each PCR reaction contained 1 μg of cDNA samples, 200 nM gene-specific primers (S2 Table) and 10 μl of 2 × SYBR Green Master Mix Reagent (Applied Biosystems). Expression levels of all genes were normalized to the levels of sigma A gene transcripts and are shown as fold change in hypoxic compared with aerobic cultures. The degrees of change in expression level were calculated using the 2^-ΔΔCt^ method [18].

### Cloning, expression and purification of CitE protein and its mutants

The *citE* gene was amplified using appropriate primers (S1 Table) from genomic DNA of *M*. *tuberculosis* H37Rv, and then cloned into the pET28a vector to produce recombinant plasmid. *E*. *coli* BL21 (DE3) cell was used to express recombinant protein. Recombinant expression strain was grown in 1 liter LB medium to an OD_600_ of 0.6 at 37 °C, and then induced by the addition of 1 mM IPTG at 16 °C for 12 h. His-tagged proteins were purified on affinity columns as described previously [18]. The purified protein elution was dialyzed against buffer (100 mM Tris–HCl (pH 7.4), 500 mM NaCl and 10% glycerol) for 2 hours and stored at -80 °C until further use. The purity of the protein was confirmed by SDS-PAGE analysis and the concentration of the protein was estimated by Coomassie Brilliant Blue assay.

As for CitE mutants in its key amino acid residues, site-directed mutations were introduced into the selected sites by overlap PCR by their respective primers (S1 Table) [19]. All mutant fragments were cloned into pET28a vector, and sequenced to confirm the success of mutant. Mutant proteins were expressed and purified according to previous method.

### Enzyme activity analysis of CitE protein

Enzyme activity of CitE protein was carried out through MDH coupled assay according to previous reports [20, 21]. The reaction velocity was determined by measuring the decrease in absorbance at 340 nm resulting from the oxidation of NADH, which was consumed accompanying with the reduction of oxaloacetic acid, one of products of CitE protein, catalyzed by MDH. The enzyme reaction mixtures were added with 20 mM citrate, 5 mM CoA, 0.1 U MDH (sigma), 0.25 mM NADH and 2.43 mg/mL CitE protein in reaction buffer (200 mM HEPES-KOH (pH 8.0), 10 mM MgCl_2_), and incubated at 37 °C. The 200 μL aliquot were collected every 5 secs for the initial 25 seconds and monitored at 340 nm for the reduction of NADH. The reaction mixture without CitE protein was the negative control. One unit of CitE enzyme activity was defined as 1 mM of NADH oxidized per min with 1 mg protein. By measuring of absorbance values during the reaction, the NADH oxidation was observed and the profile became linear. The enzyme activity units of CitE protein were calculated from the consume rate of NADH. Each measurement was performed in triplicate in experiments. Statistical differences were calculated using one-way ANOVA with Dunnett’s post-tests for comparing the activity of each mutant protein to the wild-type CitE.

### The effect of metal ions on enzyme activity

To estimate the influence of metal ions on enzyme activity, the ions were pre-removed from CitE protein by dialyzing against MES buffer (20 mM MES-NaOH, pH 6.5, 2 mM EDTA and 2 mM 1,10-phenanthroline) for 24 h, and then against dialysis buffer (100 mM Tris–HCl (pH 7.4), 500 mM NaCl and 10% glycerol) for 2 h to remove the remaining EDTA and 1,10-phenanthroline [22]. The metal ion contents in the CitE protein were determined using Atomic Absorption Spectrophotometry [23]. The enzyme activities of CitE protein and apo-CitE were compared with each other by MDH coupled assay. Furthermore, apo-CitE protein was titrated by additional metal ions to determine their effect on enzyme activity. The experiments were conducted in triplicate. Statistical differences were calculated using one-way ANOVA with Dunnett’s post-tests. Wildtype and mutant CitE Holo-enzymes were used as control groups.

### Construction of the citE deletion mutant of M. bovis and Southern blot analysis

Knockout of the *citE* gene in *M*. *bovis* was performed as described previously [24]. A pMind-derived suicide plasmid was constructed according to previous method and a *lacZ* gene was inserted into this plasmid as a blue-white selection marker. The recombinant plasmid was transformed into *M*. *bovis* BCG strain and selected on Middlebrook 7H10 solid medium (Difco) with 100 mg/mL hygromycin. Deletion of the *citE* gene was confirmed by Southern blot analysis [25]. The probe used for hybridization consisted of a 300-bp fragment amplified from the upstream region of the *citE* gene using appropriate primers (S2 Table).

### Construction of the overexpression strain and citE complementation strains of M. bovis

CitE gene was amplified from *M*. *bovis* genomic DNA by their respective primers (S2 Table). The target PCR fragment was ligated to the pMV261 vector and then transformed into *M*. *bovis*. To overproduce in *M*. *bovis*, *citE* gene was inserted downstream of the *hsp60* promotor of pMV261 [25]. Furthermore, the *citE* gene was also cloned into a pMV361 vector [25] for complementing it in *citE*-deleted *M*. *bovis* strains. The recombinant plasmids were electrophorated into *M*. *bovis* and selected on 7H10 medium containing 30 mg/mL kanamycin. The presence of the correct gene sequence in the plasmid construct was verified by DNA sequencing.

### Bacterial invasion and intracellular survival assays

The intracellular survival assays were performed by using murine macrophage RAW264.7 as previously described [26] with modifications. Briefly, prior to infection, cells were cultured in DMEM medium supplemented with 10% FBS and grown into a density of 1×10^6^ cells. The cell monolayers were infected with *M*. *bovis* BCG at a multiple of infection (MOI) of 35. After infection, the cells were washed and overlaid with a DMEM medium containing penicillin and streptomycin (100 μg/mL) to kill extracellular bacteria for 1 h. To release bacteria from macrophages for subsequent survival rate determination, cells were lysed for 10 min at room temperature in 500 μL Tween 20 at indicated time points (0 h, 4 h, 24h and 48 h after infection). The numbers of released viable bacteria were determined on 7H10 agar plates for colony count and the survival rate was calculated [27].

## Results

### Hypoxic conditions induce the expression of key rTCA cycle genes in both M. bovis BCG and M. tuberculosis H37Ra

**Fig 1:**
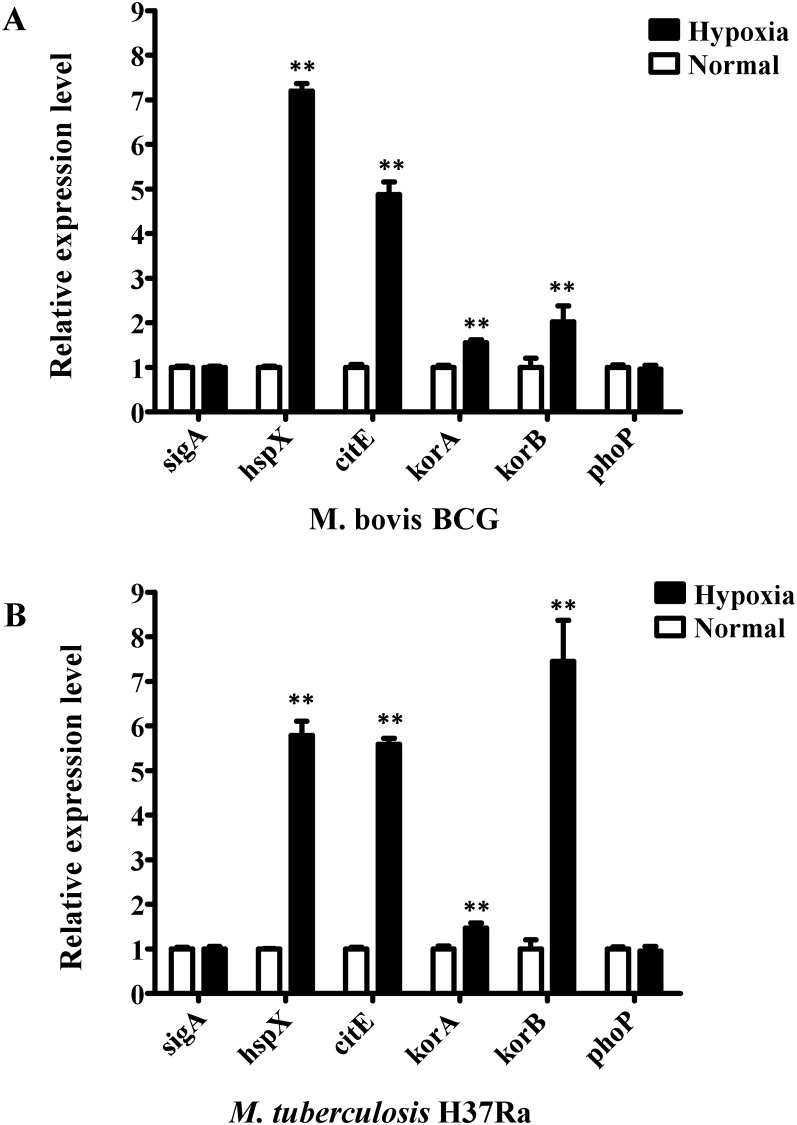
Differential expression assays of genes in normal and hypoxic cultured *M*. *bovis* BCG and *M*. *tuberculosis* H37Ra strains. Relative expression levels of *citE* gene and genes related to the rTCA cycle were analyzed in *M*. *bovis* BCG strains (A) and *M*. *tuberculosis* H37Ra strains (B) under normal and hypoxic culture conditions. The *sigA* gene was used as an invariant transcript, and an unrelated Rv0757 gene or BCG_0809 was used as a negative control. Data were analyzed by 2^-ΔΔCt^ method. The diagram shows the mean values of mRNA expressive variations from three biological replicates ± standard deviations. Relative expression data were analyzed for statistical significance by the one-way ANOVA with GraphPad Prism. Data significance (**, *P* <0.01) was calculated by single effect analysis.

The expression of three key rTCA cycle genes (*citE*, *korA*, and *korB*) was assessed in *M*. *bovis* BCG and *M*. *tuberculosis* H37Ra under hypoxic stress conditions. As shown in Fig 1A and 1B, the expression levels of all target genes in both *Mycobacterium* species were significantly upregulated compared with the control under hypoxic stress conditions. A gene activated by *dosR* under hypoxia stress, *hspX*, was used as a positive control [28] and exhibited a significant increase in expression. By contrast, the expression of negative control gene *phoP* was not significantly altered. This finding indicated that the rTCA cycle played a major role in cellular metabolism under hypoxic stress conditions.

### MtbCitE is a citrate lyase

**Fig 2:**
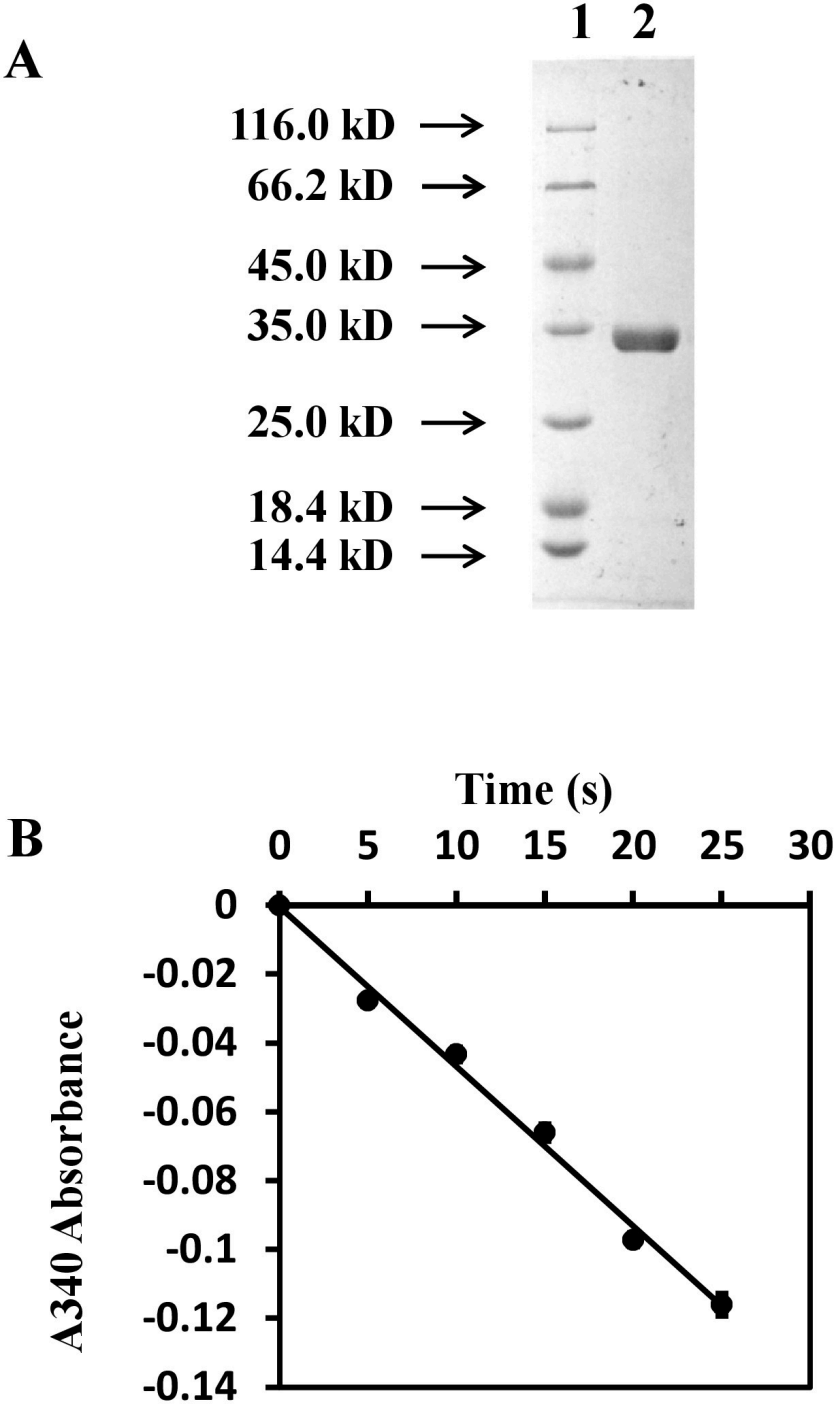
Purification and functional identification of MtbCitE protein. Identification of citrate cleavage activity embraced by MtbCitE. The measurement of citrate cleavage activity was coupled with MDH assay. Oxaloacetate, product of CitE, is used as the substrate of MDH combined with oxidation of NADH, which was monitored spectrophotometrically at 340 nm. The values presented are the average values of three independent experiments, and 0.49 mg of CitE was added in the reaction mix. The line in the graph shows the trend of NADH oxidation rate, which represents the enzyme activity of MtbCitE.

The purified MtbCitE protein was found in the molecular weight range 25.0–35.0 kDa when assessed by SDS-PAGE (Fig 2A). Chemical cross-linking assay proved that MtbCitE protein can form trimer structure (S1 Fig). Following the expression and purification of the MtbCitE protein, the activity of the purified MtbCitE was measured. As shown in Fig 2B, a gradual decline in the absorbance ratio was observed with increasing reaction time. This result suggested that purified CitE protein might degrade citrate to citryl-CoA and oxaloacetic acid, which could be further reduced by malate dehydrogenase with the addition of NADH, leading to a decrease in the absorbance ratio. Thus, we predicted that purified MtbCitE had citrate lyase activity.

### CitE contains conserved amino acid residues

**Fig 3:**
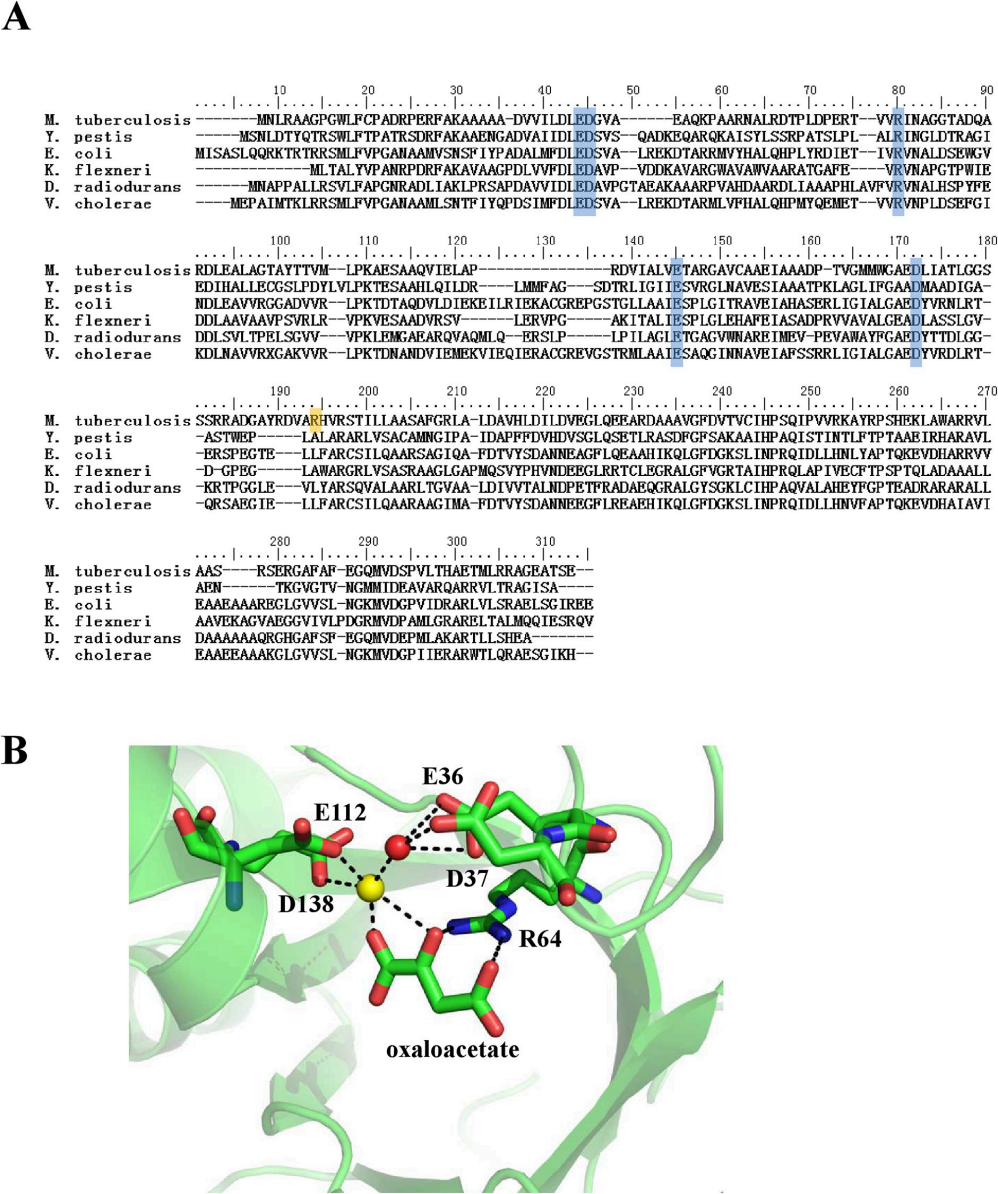
Selection of conservedconversed residues in CitE catalytic site. (A) Multiple sequence alignment between MtbCitE (citrate lyase β-subunit) and citrate lyase β-subunit from *Y*. *pestis*, *E*. *coli*, *K*. *flexneri*, *D*. *radiodurans* and *V*. *cholerae*. The conserved residues in these species are highlighted in blue; the residue involved in ATP binding is highlighted in orange. The amino acid sequence alignment was analyzed using CLUSTALW. (B) Cartoon diagram of MtbCitE with magnesium ion and oxaloacetate. The illustration was generated with structure 1Z6K deposited in the PDB by PYMOL. Magnesium ion and water molecule are shown as a yellow sphere and a red sphere respectively. The residues involved in binding Mg^2+^ and oxaloacetate are shown in a stick representation with differently colored atoms. The hydrogen bonds between atoms are shown with broken black lines.

We then compared the amino acid sequence of CitE from *M*. *tuberculosis* H37Rv with those from *Yersinia pestis*, *Escherichia*. *coli*, and *Shigella flexneri* to identify conserved residues (Fig 3A). Given that several amino acid residues were conserved, only those that were likely to participate in enzyme activity and metal ion binding, as suggested by previous studies [15, 29], were examined further. On the basis of a previous report, Asp37 and Asp138 were identified as likely to participate in enzyme activity. Located within the probable catalytic site of CitE, these two hydrophilic residues belong to a hydrophobic cavity formed by the triosephosphate isomerase (TIM) β-barrel structure of the enzyme (Fig 3B) (15). Arg64, another conserved residue, may be involved in citrate cleavage by binding to oxaloacetate (Fig 3B).

CitE is thought to be a Mg^2+^-dependent citrate lyase (9). As shown in Fig 3B, Glu36, Glu112, and Asp138 are likely involved in the magnesium ion interaction. Glu112 and Asp138 may bind directly to Mg^2+^, whereas Glu36 may interact with Mg^2+^ through a water molecule. In addition, the 50 amino acids at the C-terminus of MtbCitE were predicted to constitute a nonspecific secondary structure that becomes disordered in solution, although its function remains unclear.^14^ Thus, site-directed mutagenesis was carried out to investigate the functions of the selected amino acids, whereas the truncated protein CitEΔC50 was constructed to identify the functional role of the 50 amino acids at the C-terminus of CitE.

### Glu36, Arg64, Glu112, and Asp138 are essential for the citrate lyase activity of MtbCitE

**Fig 4:**
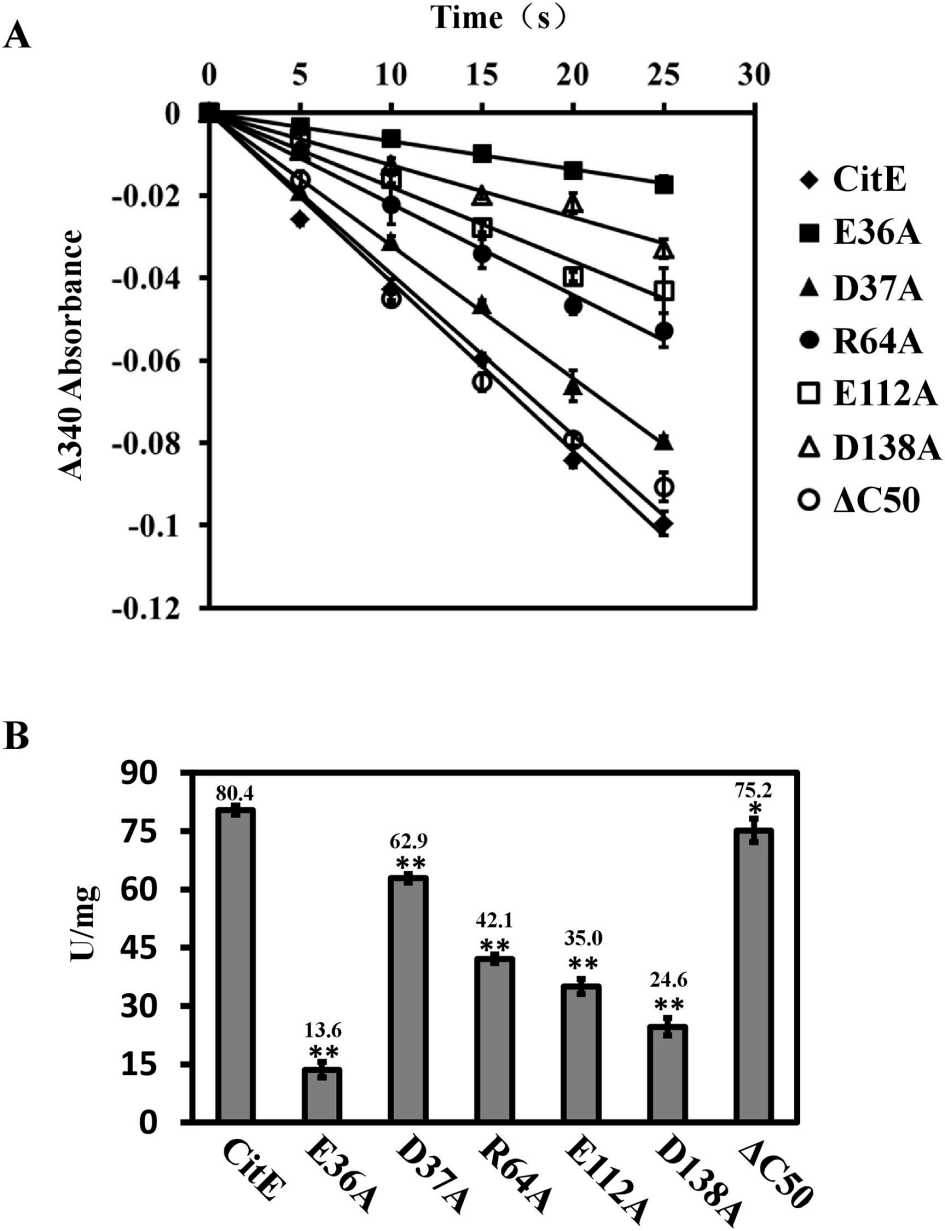
Comparison of citrate cleavage activity between wild-type MtbCitE and its mutants. (A) The linear plot reveals the trends of citrate cleavage by wild-type MtbCitE and its mutants. The line in the graph shows the trend of NADH oxidation rate, which represents the enzyme activity of MtbCitE and its mutants. Diamond, wild-type CitE protein; close square, E36A; close triangle, D37A; close circle, R64A; open square, E112A; open triangle, D138A; open circle, ΔC50). The diagram shows the mean values of A340 absorbance from three independent replicates ± standard deviations. (B) The bar chart reveals the citrate cleavage activity units of wild-type MtbCitE and its mutants. The values presented are the average values of three independent experiments, and the error bars represent the variant range of the data derived from three replicates. The *P*-values of the results were calculated by one-way ANOVA. A significant difference between two proteins was indicated by an asterisk (*, *P*<0.05) or a double asterisk (**, *P*<0.01) in the figure.

The specific enzyme activity of each of the MtbCitE mutants was compared with that of the wild-type MtbCitE protein. As shown in Fig 4, compared with wild-type CitE, CitE mutants E36A, R64A, E112A, and D138A exhibited a significant decrease in citrate cleavage activity, with E36A demonstrating the greatest decrease. By contrast, no obvious change in citrate cleavage activity was observed for mutant D37A compared with wild-type CitE. These results indicated that Glu36, Arg64, Glu112, and Asp138 were likely to be the key amino acid residues for MtbCitE citrate cleavage activity.

Truncated protein CitEΔC50 showed only a ~6% decrease in citrate cleavage activity compared with wild-type CitE, suggesting that the 50 amino acids at the C-terminus were not essential for the enzymatic activity of MtbCitE. Thus, the function of this region remains unclear.

### MtbCitE activity depends on Mg^2+^

**Fig 5:**
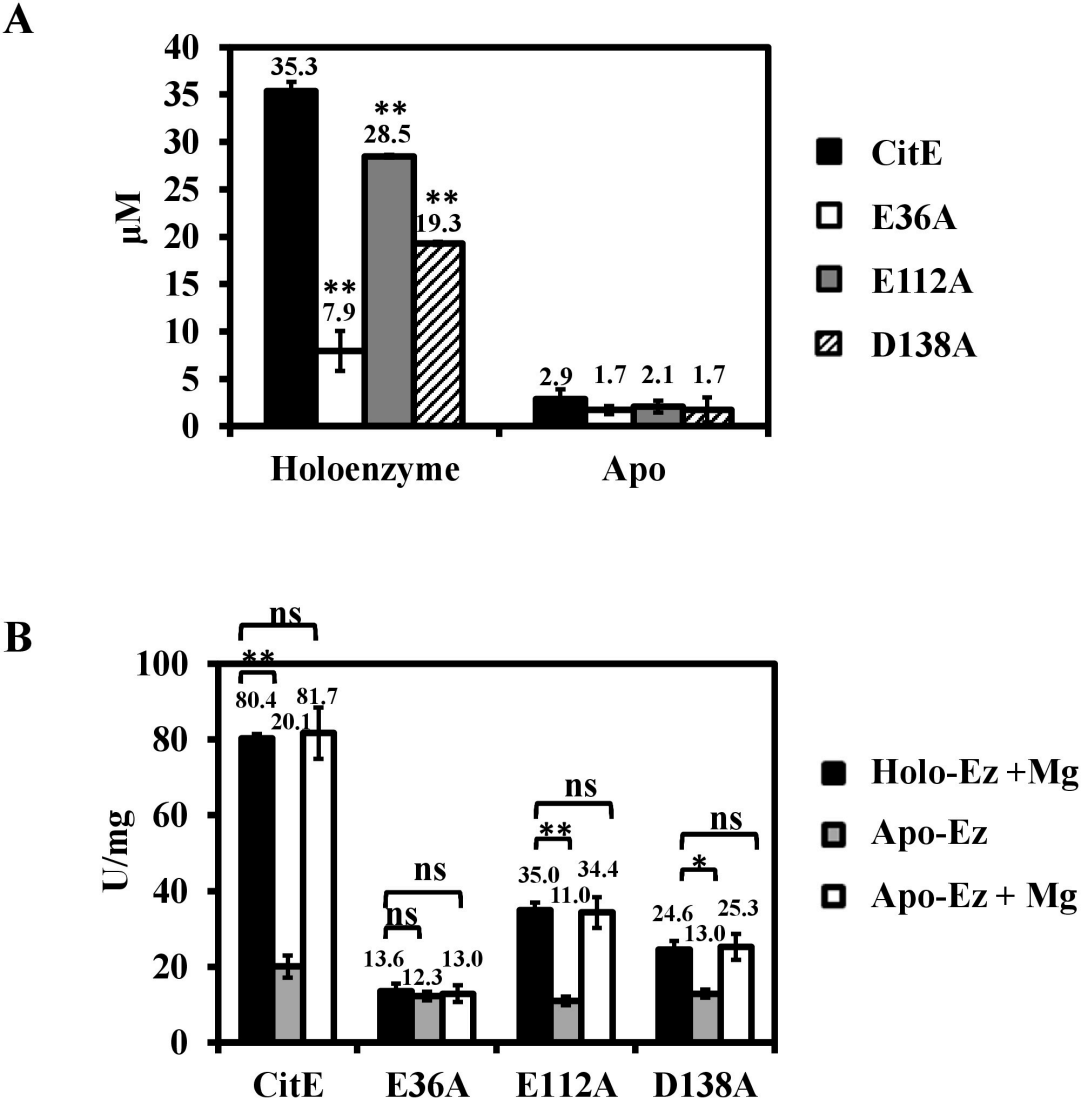
Effect of Mg^2+^ on MtbCitE activity. (A) The Mg^2+^ concentrations of wild-type MtbCitE and its mutants. Mg^2+^ concentrations in the samples were detected by atomic absorption spectrophotometry. The protein concentration used in this assay was 100 μM. Purified proteins (800 mL 2.0 mg/mL) were digested with nitric acid (200 mL) and diluted to 4 mL. To remove ions from the wild-type proteins and obtain apo-CitE proteins (magnesium free form of CitE proteins), the purified CitE proteins were dialyzed for 24 h against the MES buffer. The one-way ANOVO of independent experiments was used for statistical analysis. The differences between the Mg2+ contents in mutant and wild-type CitE proteins were calculated using Dunnett’s post-tests. The Mg2+ concentrations of wild-type CitE protein was used as control groups. (B) Comparison of citrate cleavage activity of wild-type MtbCitE and its mutants in the presence and absence of Mg^2+^. Mg^2+^ was pre-removed from the enzyme protein by dialysis. The black bars represent holo-enzyme with 10 mM Mg^2+^, grey bars represent the apo-enzyme alone, and white bars represent apo-enzyme added with 10 mM Mg^2+^. The bar values represent the averages of three independent experiments, and the error bars represent the variant range of the data derived from the replicates. The *P*-values of the data were calculated by one-way ANOVA. Dunnett’s post-tests were used to analyze the differential significance between protein activities (*, *P* <0.05; **, *P* <0.01; ns, *P*>0.05).

The effect of Mg^2+^ on citrate cleavage activity was systematically investigated to determine whether the activity of CitE is Mg^2+^ dependent. Apo-CitE was obtained by pre-removing Mg^2+^ from the protein by dialysis, and atomic absorption spectrophotometry confirmed that magnesium was completely removed (Fig 5A). Compared with wild-type MtbCitE, apo-CitE demonstrated a 75% decrease in citrate cleavage activity (Fig 5B). In addition, activity was restored when Mg^2+^ was added to the apo-CitE reaction mixtures. These results suggested that CitE was a Mg^2+^-dependent protein.

The same method was used to examine the effects of magnesium on CitE mutants containing substitutions at predicted Mg^2+^ binding sites (E36A, E112A, and D138A) (14). As shown in Fig 5A, mutant E36A showed an 80% decrease in Mg^2+^ binding ability compared with wild-type CitE. Combined with the observed 83% reduction in citrate cleavage activity of E36A (Figs 4 and 5B), these results suggested that Glu36 was essential for Mg^2+^ binding. Decreases in binding were also observed for mutants E112A and D138A (~60% and 70%, respectively), although they were not as dramatic as that of E36A. These findings indicated that Glu112 and Asp138 could also bind Mg^2+^, and they may reinforce the Mg^2+^-binding activity of MtbCitE by assisting Glu36.

### ATP inhibits the citrate cleavage activity of MtbCitE

**Fig 6:**
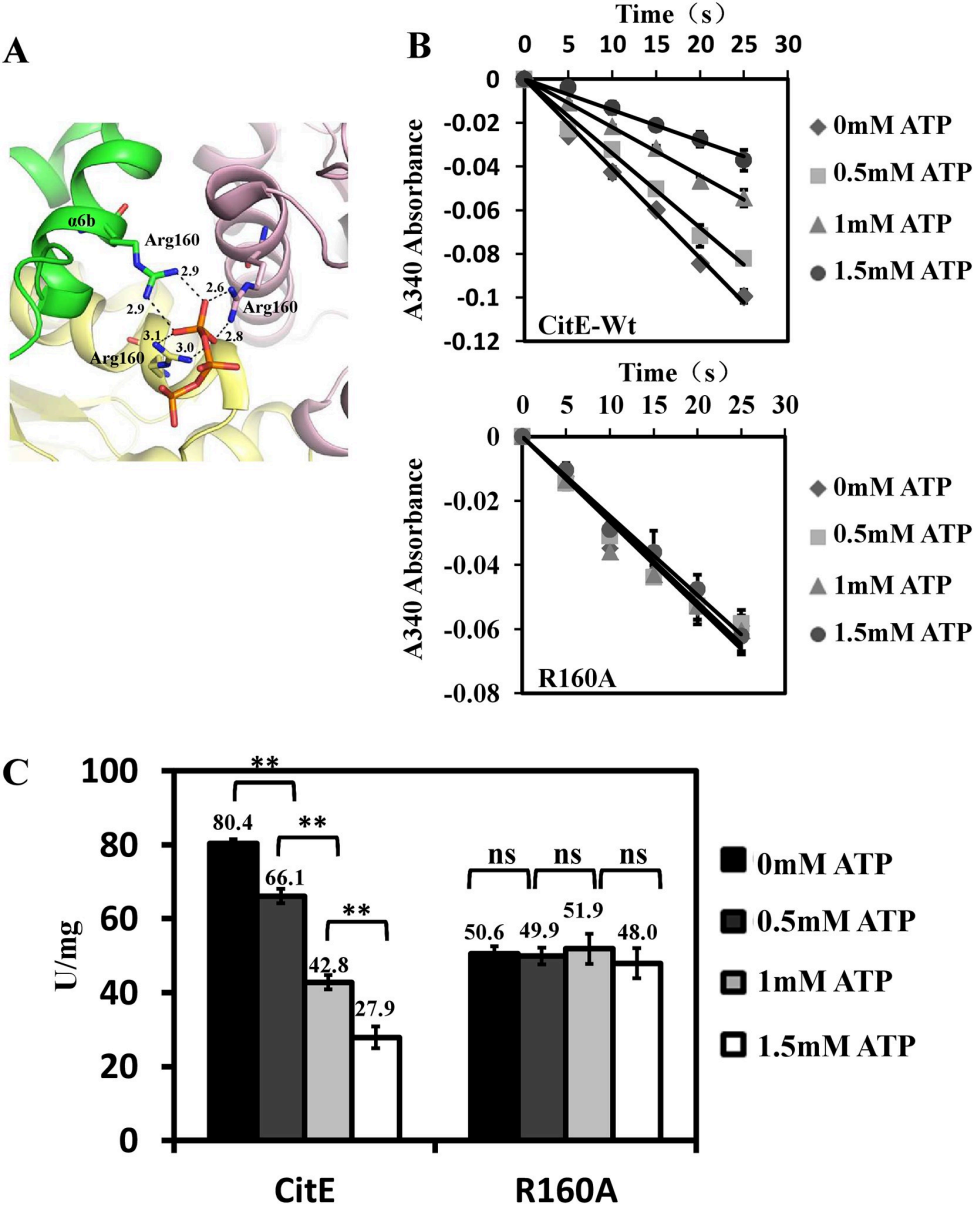
Effect of ATP molecule on MtbCitE activity. (A) Cartoon diagram of MtbCitE trimer with an ATP molecule. The graph was created with PDB entry 1U5V by PYMOL. Protein polymer was colored differently by monomers. The phosphate group of the ATP molecule and the residue Arg160 involved with ATP binding are shown in a stick form and colored differently by atoms. The hydrogen bonds between the oxygen atoms of ATP molecule and nitrogen atoms of residues are shown with broken black lines and the distance between the atoms is indicated. (B) Comparison of citrate cleavage activity of wild-type MtbCitE (top) and mutant R160A (bottom) with increasing amount of ATP. ATP molecule at a final concentration ranging from 0 mM to 1.5 mM was added to reaction mixtures. Diamond, 0 mM; square, 0.5 mM; triangle, 1 mM; circle, 1.5 mM. The diagram shows the mean values of A340 absorbance from three independent replicates ± standard deviations. (C) The bar chart reveals citrate cleavage activity units of wild-type MtbCitE and mutant R160A with increasing amount of ATP. The bar heights represent the mean value of enzyme activity units of three independent experiments, and the error bars represent the variant range of the data derived from the replicates. Significant difference of data was indicated as the *P*-values of the data were calculated by one-way ANOVA (**, *P* <0.01; ns, *P* >0.05). The wild-type and mutant CitE activities in the absence of ATP were used as control groups. Dunnett’s post-tests were used to analyze the differential significance between protein activities.

To determine the effects of ATP on the function of MtbCitE, increasing concentrations of ATP were added to reaction mixtures, and citrate cleavage activity was examined. A gradual decrease in citrate cleavage was observed with increasing concentrations of ATP (Fig 6B and 6C), thereby indicating that the citrate cleavage activity of MtbCitE was inhibited by ATP. Furthermore, by analyzing the data for Protein Data Bank entry 1U5V, we determined that the CitE-ATP complex was formed in solution. Arg160 may coordinate this interaction through binding of the nitrogen atoms of its two NH_2_ groups to the γ-phosphate group of the ATP molecule (Fig 6A). No significant change in the citrate cleavage activity of R160A was observed following the addition of increasing concentrations of ATP (Fig 6B and 6C). Arg160 may be the key amino acid residue in the interaction of CitE with ATP.

### CitE contributes to the survival of M. bovis BCG under hypoxic stress

**Fig 7:**
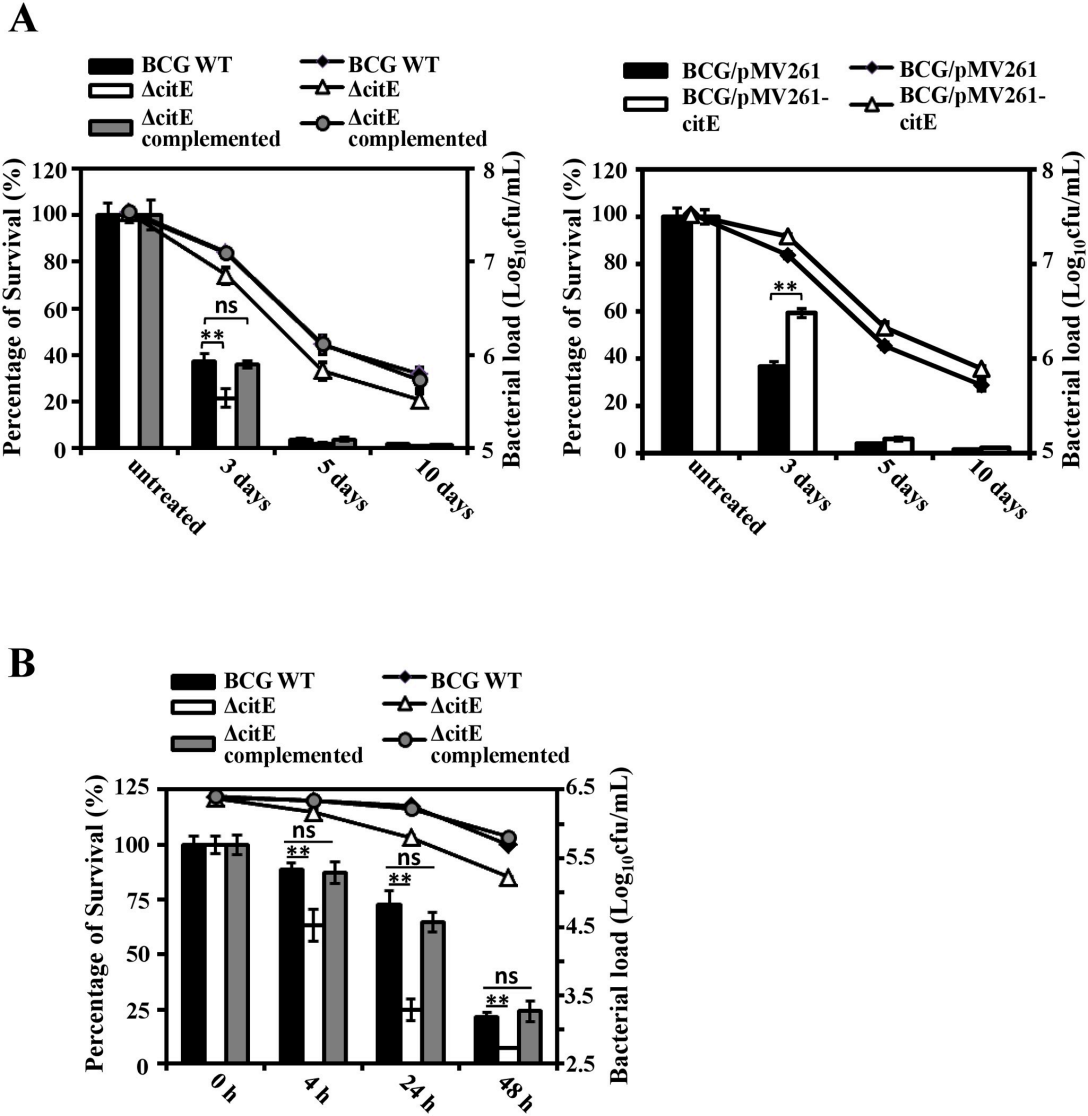
Effect of hypoxic condition on the survival rate of mycobacterial strains. (A) *M*. *bovis* BCG strains were treated with hypoxic condition for 3 days, 5 days or 10 days before samples were harvested, and bacterial viability was assessed by CFU assay. The bar graph indicates the quantification of live cells relative to untreated bacteria. The diagram shows the mean values of the percentage of surviving bacteria compared with the untreated group from three biological replicates, and the error bars represent standard deviations. And curves represent the mean value of bacterial loads (log_10_cfu/mL) of different BCG strains with standard errors. Values for normalized bacterial loads are indicated on the right y-axis. (B) Intracellular survival assays. *M*. *bovis* BCG wild-type (black bar), *citE*-deleted mutant (white bar) and complemented mutant (grey bar) strains were used to infect macrophage RAW264.7. After 4 h, 24 h and 48 h infection, the intracellular survival rates of three mycobacterial strains were determined. The bar graph indicates the percentage of viable intracellular bacteria over total infected bacteria. Bacterial loads are indicated on the right y-axis. Results are presented from three independent experiments and error bars represent standard errors of mean values (SEM). Statistical differences were calculated using one-way ANOVA with Dunnett’s post-tests for comparing each treatment group to the untreated control samples. **, *P* < 0.01; ns, *P* > 0.05.

A *citE*-deleted *M*. *bovis* BCG strain (Δ*citE*) was generated by allelic replacement (S2 Fig), and a complemented mutant strain was constructed by transformation of pMV361-*citE* into the Δ*citE* strain. The survival rates of the deletion mutant, complemented mutant, and wild-type strain were then examined under hypoxic conditions. As shown in Fig 7A, no substantial difference in CFU was observed among the wild-type, Δ*citE*, and complemented mutant strains under normal growth conditions. However, we observed a significant difference (*P* = 0.00656) between the bacterial load of wild-type strain (1.24×10^7^ cfu/mL) and Δ*citE* strain (0.72×10^7^ cfu/mL) after 72 hours of hypoxic culture. Consistently, the survival rate of the Δ*citE* mutant was appreciably lower than that of the wild-type strain under hypoxic conditions, whereas the survival rate of the complemented mutant strain was similar to that of the wild-type strain BCG. In addition, the BCG strain overexpressing *citE* from pMV261 had a higher survival rate than the wild-type strain containing the empty pMV261 vector (Fig 7A, right panel). Thus, *citE* has an effect on the survival of BCG under hypoxic conditions.

### CitE contributes to intracellular survival of M. bovis BCG

Next, we determined the contribution of CitE to the mycobacterial survival in infected macrophage. As shown in Fig 7B, after 4 hours of infection, we observed a significant difference (*P* = 0.00500) between the survival rate of Δ*citE* strain (63.5% ± 7.2%) and wild-type strain (88.7% ± 2.9%). The difference became more obvious (P = 0.00057) if we further determined their survival rates after a 48 h infection, and the wild-type BCG strain obtained a 3-fold higher survival rate (21.4% ± 2.5%) than the *citE*-deleted strain (7.0% ± 0.2%) (Fig 7B). Strikingly, no significant survival difference was observed between complemented strain and wild-type BCG under a similar condition. These data suggested that CitE contributes to intracellular survival of *M*. *bovis* BCG.

## Discussion

According to the previous study by Arora G. et al, MtbCitE protein did not show citrate cleavage activity (15). But in this study, MtbCitE was confirmed, alone and directly, catalyzed the cleavage of citrate to acetyl-CoA and oxaloacetate. For the reason, we suggest that MtbCitE possesses lower activity as higher concentration protein was used when investigating the citrate cleavage activity of MtbCitE. Furthermore, we showed that MtbCitE was Mg^2+^ dependent, and ATP could inhibit citrate cleavage activity. These findings indicated that the biochemical function of MtbCitE differed from that of other bacterial citrate lyases, including those from *Escherichia coli* [30], *Klebsiella pneumoniae* [31], and *Leuconostoc paramesenteroides* [32]. These previously described citrate lyase enzymes consist of an ATP-independent complex composed of six copies of each subunit, α, β, and γ. The β-subunit of these citrate lyase complexes is homologous to CitE, but cannot catalyze citrate cleavage by itself. Thus, the function of MtbCitE differs from that of other bacterial pathogens.

Despite the functional differences between MtbCitE and other bacterial CitE enzymes, amino acid sequence comparison identified the conserved residues involved in catalytic activity (Fig 3A). Site-directed mutagenesis aimed at identifying specific residues involved in the activity of MtbCitE demonstrated that all mutants, except D37A, presented decreased citrate catalysis activity compared with wild-type CitE. Asp138, targeted in mutant D138A, is located within the predicted catalytic site of CitE that forms a hydrophobic cavity. The significant decrease in catalytic activity of D138A confirmed that hydrophilic residue Asp138 was part of the active site of CitE. Arg64 was also predicted to play a role in catalysis through its ability to bind oxaloacetate. The decrease in citrate cleavage activity of mutant R64A indicated that the citrate catalytic activity of CitE was also partly dependent on its enzyme-product interaction ability. Furthermore, as a Mg^2+^-dependent citrate lyase, amino acid residues relating to metal ion binding are particularly important for CitE activity. In this study, a complete loss of enzymatic activity was observed in mutant E36A, which could not be restored by the addition of magnesium ions. Therefore, Glu36 was essential for the Mg^2+^ binding ability of CitE.

**Fig 8:**
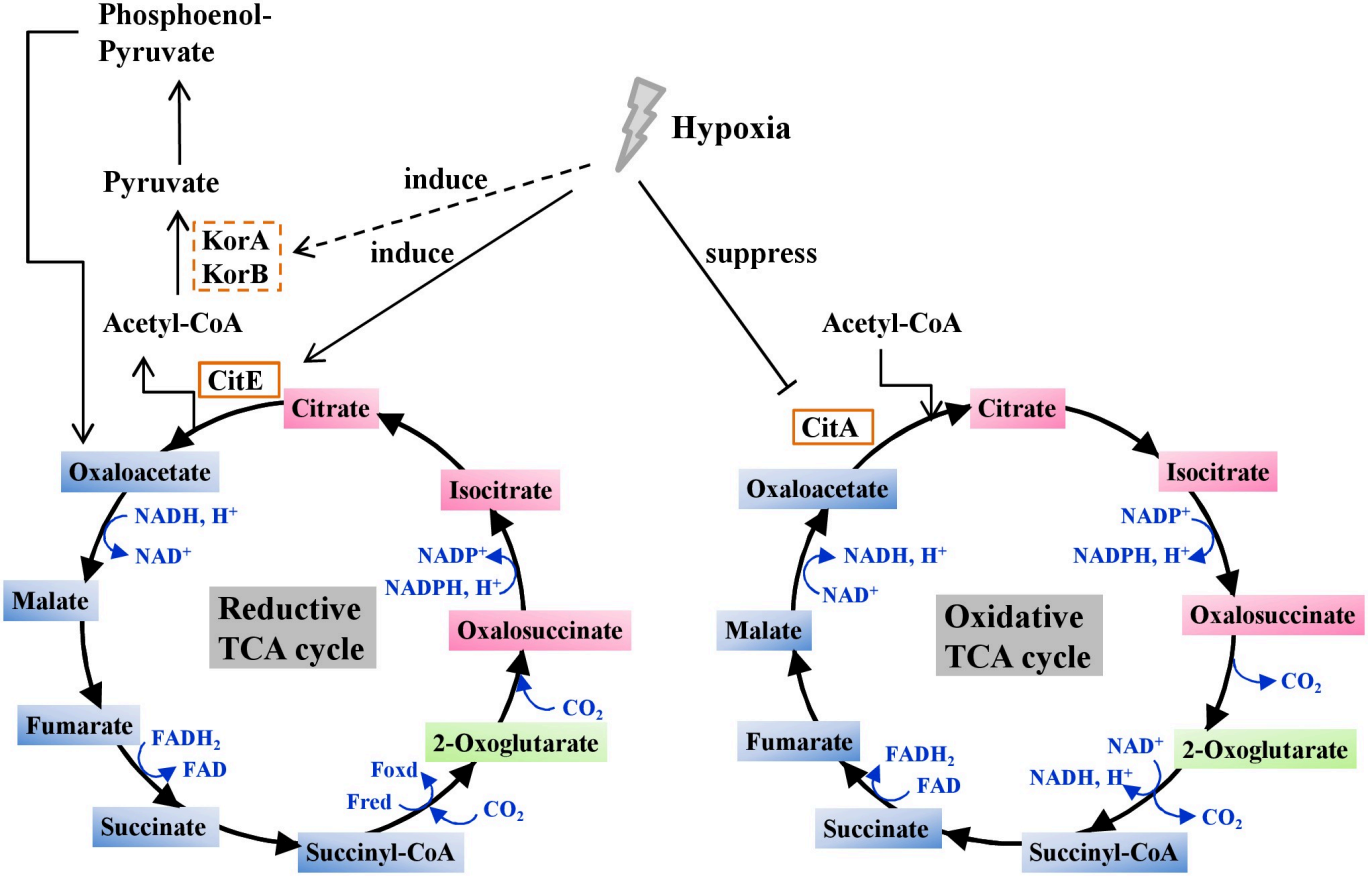
Schematic diagram summarizing the role of hypoxia in regulating energy metabolism in *M*. *tuberculosis*. Under hypoxic conditions, *M*. *tuberculosis* has an increased expression level of *citE*, *korA* and *korB*, which have important roles in reductive TCA cycle. In contrast, citrate synthetase *citA* which functional in oxidative TCA cycle has a decreased expression level in hypoxic *M*. *tuberculosis*. Hypoxia also results in reduction of intracellular ATP level and accumulation of reduced cofactors in *M*. *tuberculosis*.

CitE has been confirmed as an ATP-independent citrate lyase in various other bacteria [30–32]. Unexpectedly, in the present study, we found that ATP inhibited the citrate cleavage activity of CitE. The amino acid residue Arg160 was further characterized as a potential ATP binding site. Notably, *M*. *tuberculosis* CitE exhibited a unique ATP-binding specificity, and the Arg160 residue was not conserved in several CitE-like enzymes (Fig 3A). Consistently, when comparing the structure of *Rhodobacter sphaeroides* MCL (4L9Y) and *M*. *tuberculosis* CitE (1U5H; S4 Fig), R160 was found to be outside the conserved domain, although two enzymes showed a similar canonical TIM barrel structure. Therefore, in the present study, we provided data to show that M. tuberculosis CitE was an ATP-sensitive citrate cleavage enzyme, although ATP was not necessary for the activity. This finding supports a model the amount of ATP produced by *M*. *tuberculosis* controls whether citrate is funneled towards the TCA cycle or rTCA cycle (Fig 8). Under optimal growth conditions, *M*. *tuberculosis* triggers the TCA cycle to provide more energy for its growth, whereas the rTCA cycle is inhibited because CitE is inactivated by the abundance of ATP molecules. However, when *M*. *tuberculosis* is subjected to unfavorable growth conditions, such as the changes in oxygen tension that occur within a host [33], the bacterium has to decrease its energy metabolism and produce a smaller amount of ATP (S5 Fig). Under these conditions, CitE activity remains high, and citrate will be metabolized to oxaloacetate and citryl-CoA. These molecules are precursors for fatty acid metabolism and cholesterol biosynthesis, which are important steps in improving the virulence of *M*. *tuberculosis* by thickening the cell wall. A different adaptation to hypoxic stress between *M*. *tuberculosis* and *M*. *bovis* BCG was previously reported [34], which may account in part for virulence differences between these two strains.

In the present study, the expression levels of genes coding for citrate lyase and 2-oxoglutarate synthase in *M*. *tuberculosis* significantly improved under hypoxic conditions (Fig 1), which is consistent with the result acquired by Arora G. et al (15). The rTCA cycle has the remarkable ability to fix carbon by sharing enzymes from the TCA cycle when *M*. *tuberculosis* is subjected to oxygen-limiting microaerophilic and anaerobic conditions (6). Citrate lyase and 2-oxoglutarate synthase are two key enzymes of the rTCA cycle. Therefore, our findings implied that the rTCA cycle dominated the TCA cycle under anaerobic conditions. This assumption was consistent with previous observations. For example, the rTCA cycle was proposed to be upregulated under hypoxic conditions, which further resulted in the significant accumulation and secretion of succinate in *M*. *tuberculosis* [4, 35]. Our findings, together with previous data, support the idea that *M*. *tuberculosis* could survive under hypoxic conditions by altering its mode of metabolism from an oxidative direction to a reductive direction.

In conclusion, MtbCitE is a Mg^2+^-dependent protein with citrate cleavage activity. The relationship between MtbCitE and ATP may be an important factor contributing to the survival of the pathogen under hypoxic conditions. Thus, MtbCitE plays important roles in the persistent growth of *M*. *tuberculosis* in hostile environments.

## Supporting information

S1 Raw images (PDF)

S1 Table Strains and plasmids used in this study. (DOCX)

S2 Table Primers used in this study. (DOCX)

S3 Table Primers for quantitative real-time PCR (qRT-PCR). (DOCX)

S1 Fig Oligomeric status of MtbCitE protein. Oligomeric status of the CitE protein was detected by Chemical Cross-Linking assay according to previous reports. The 20 μl reaction mixes, including 10 mM CitE protein, 4 μM Disuccinimidyl suberate (DSS) and cross-linking buffer (100 mM NaH2PO4 pH 8.0 and 150 mM NaCl), were incubated at room temperature. Half an hour later, the reaction was stopped by incubating with standard SDS-PAGE sample buffer at 100 °C for 5 min, and then analyzed by SDS-PAGE.
(TIF)

S2 Fig Construction of the *M*. *bovis citE* knockout strain and Southern blotting assay. (A) The double-crossover recombinant strategy for *M*. *bovis citE* gene knockout. Upstream, 1 kb genomic DNA of citE gene upstream; Downstream, 1 kb genomic DNA of citE gene downstream; Hyg, hygromycin resistance gene. (B) A map of the recombinant vector constructed for *citE* knockout. Upstream DNA fragment was inserted between *Pac* I and *Spe* I sites of the vector; downstream was placed at *Nhe* I site of the vector; LacZ gene was put into *Pac* I site of the vector. (C) Schematic of genomic DNA from wild-type and *citE* knockout strains digested with the restriction enzyme *Pst* I. The probe is indicated with a black bar. (D) Southern blot assays. Southern blotting was performed to detect genomic DNA containing citE gene or hygromycin gene. A 300-bp probe corresponding to the digested fragment was obtained by PCR and labeled with digoxigenin dUTP (Roche, Mannheim Germany). The samples were hybridized with the external probe, and they showed changes in the size before and after recombination.
(TIF)

S3 Fig Sequence alignment between CitE from *M*. *tuberculosis* H37Rv and *M*. *bovis* BCG. The sequence alignment of amino acids shows the homology of the citE protein in TB to BCG are 100% identity.
(TIF)

S4 Fig Comparison of *R*. *sphaeroides* malyl-CoA lyases and MtbCitE in the sequence and conformation. (A) Overlay of *R*. *sphaeroides* malyl-CoA lyases and MtbCitE. MCL (PDB 4L9Y) is colored grey and CitE (PDB 1U5H) is colored in blue. R160 of MtbCitE is showed in red sticks. (B) Comparison of MCL and CitE in sequence. The residues overlaid with MCL in structure colored blue in CitE sequence. R160 of MtbCitE is indicated by red box.
(TIF)

S5 Fig ATP level of *M*. *bovis* BCG under hypoxic stress. The *M*. *bovis* BCG was cultured and collected according to the Materials and Methods.
(TIF)
