# Supplementary material for: Citrate lyase CitE in Mycobacterium tuberculosis contributes to mycobacterial survival under hypoxic conditions
Source: PLoS One. 2020 Apr 17;15(4):e0230786. doi: 10.1371/journal.pone.0230786 (PMC7164622; doi:10.1371/journal.pone.0230786)
Supplement: S1 Raw images — (PDF) [file pone.0230786.s001.pdf]

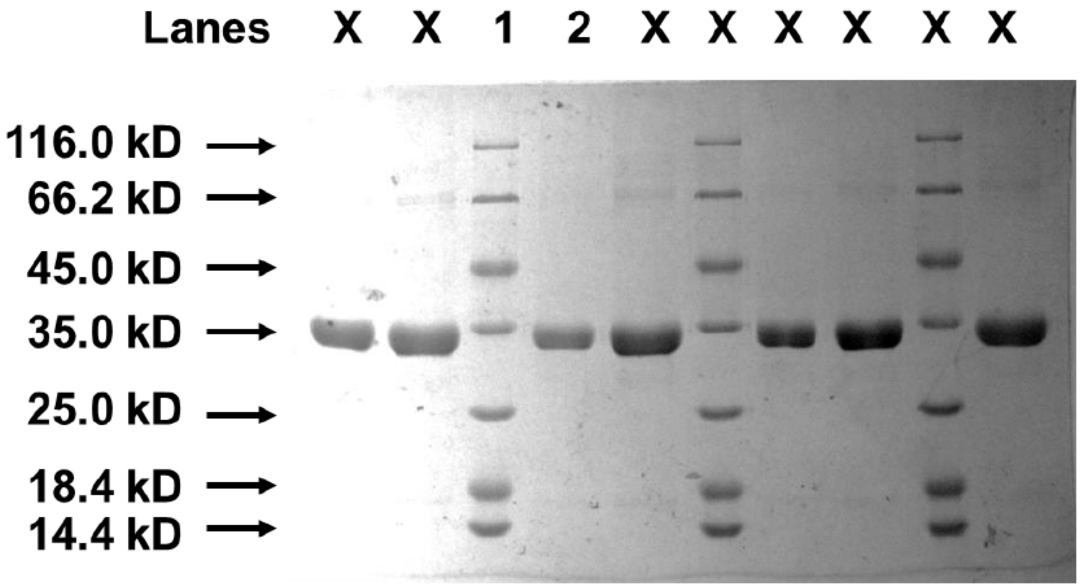

Raw images 1. SDS-PAGE. The gel image was analyzed by running a gel imaging system (GelDoc™ XR+ Gel Documentation System, Bio-Rad, USA). Lane 1, marker; Lane 2, purified MtbCitE protein.

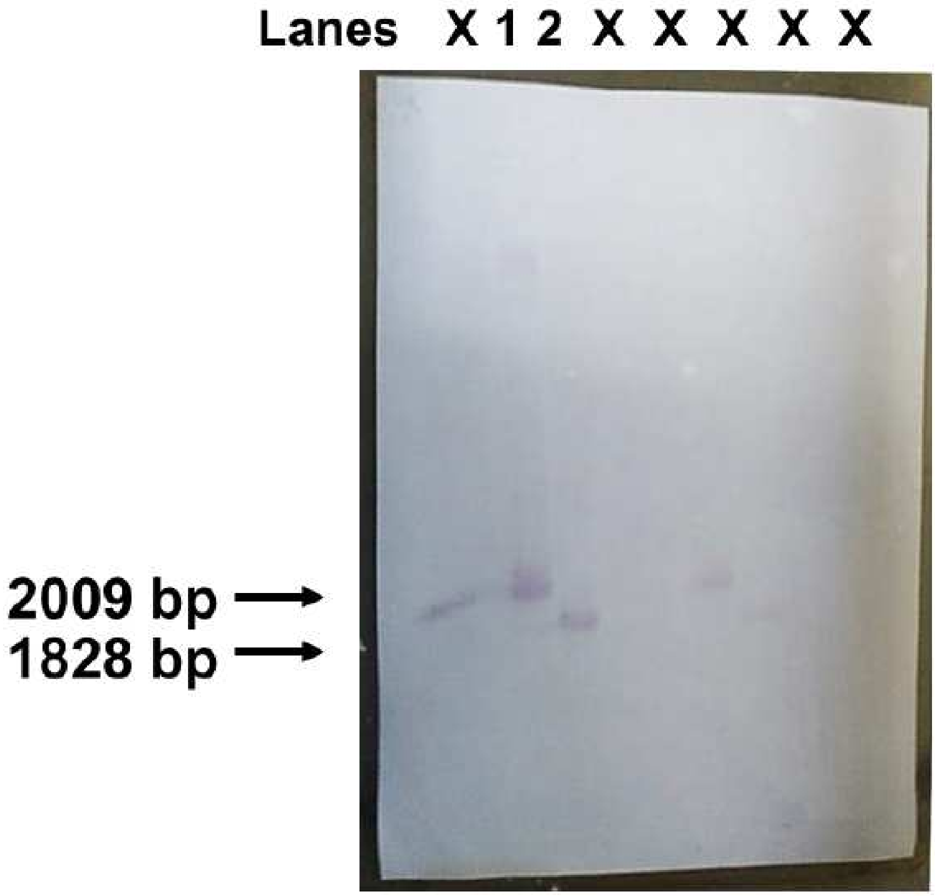

Raw images 2. Southern blot assays. The image was taken by a camera (SONY DSC-RX10). Lane 1, *BCG/WT* choromosomal DNAs digested with PstI; Lane 2, *BCG/citE::hyg* choromosomal DNAs digested with PstI.

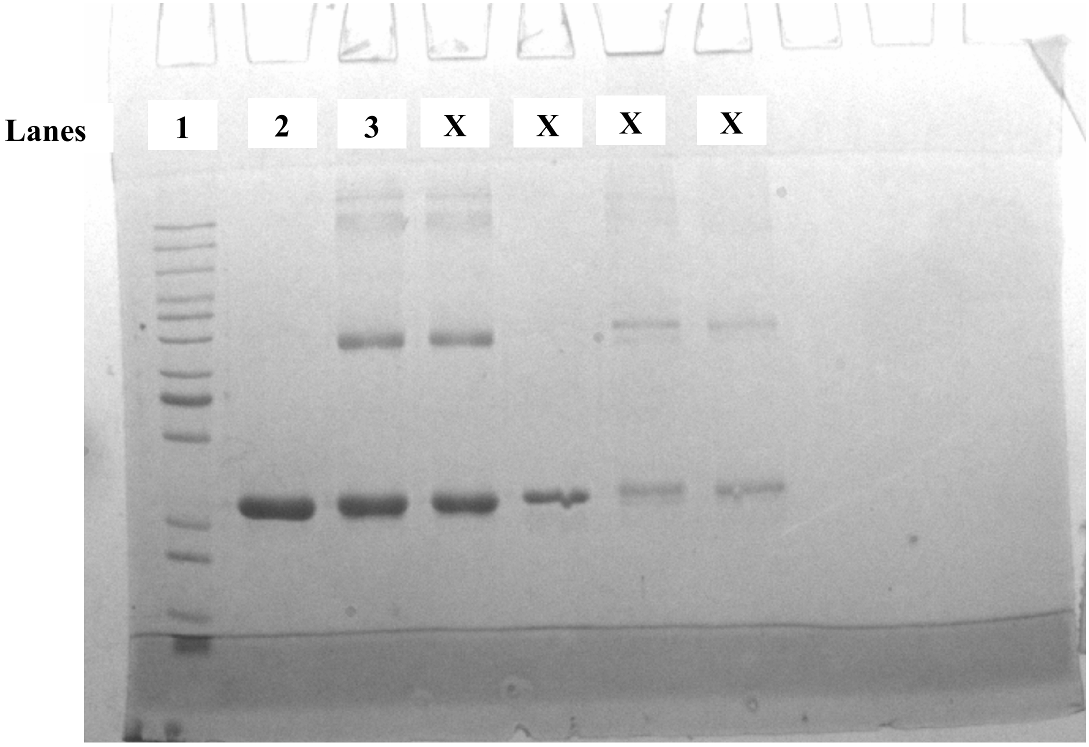

Raw images 3. Chemical Cross-Linking assay. The gel image was analyzed by running a gel imaging system (GelDocTM XR+ Gel Documentation System, Bio-Rad, USA). Lane 1, marker; Lane 2, MtbCitE protein; Lane 3, MtbCitE Protein + DSS.
