# Supplementary material for: Citrate lyase CitE in Mycobacterium tuberculosis contributes to mycobacterial survival under hypoxic conditions
Source: PLoS One. 2020 Apr 17;15(4):e0230786. doi: 10.1371/journal.pone.0230786 (PMC7164622; doi:10.1371/journal.pone.0230786)
Supplement: S1 Table — (DOCX) [file pone.0230786.s002.docx]

**Table S1 Strains and plasmids used in this study**

| Plasmid or Strain | Relevant genotype or features | Source or reference |
| --- | --- | --- |
| Strain  *E.coli* |  |  |
| DH5a | Host for plasmid construction | TaKaRa |
| BL21 | Host for overexpression | TaKaRa |
| *M. bovis* |  |  |
| BCG WT | *M. bovis* |  |
| BCG/pMV261 | *M. bovis* with pMV261 | This study |
| BCG/pMV261-B2518c | *M. bovis* with pMV261::BCG_2518c | This study |
| BCG B2518c::hyg | *M. bovis* gene *bcg_2518c* replaced by *hyg* | This study |
| BCG B2518c::hyg /pMV361-B2518c | Msm0535::*hyg* with pMV261 | This study |
| Plasmid  pET28a(+) | Kan^r^, lacZ operon, T7 promotor, His-Tag | Novagen |
| pET-CitE | CitE in *EcoR*I-*Xba*I of pET28a | This study |
| pMV261 | Kan^r^, pAL5000 replicon |  |
| pMV261-B2518c | *M. bovis* gene *bcg_2518c* in *EcoR*I-*Xba*I of pMV261 | This study |
| pMV361-B2518c | *M. bovis* gene *bcg_2518c* in *EcoR*I-*Xba*I of pMV361 | This study |
