# Supplementary material for: Citrate lyase CitE in Mycobacterium tuberculosis contributes to mycobacterial survival under hypoxic conditions
Source: PLoS One. 2020 Apr 17;15(4):e0230786. doi: 10.1371/journal.pone.0230786 (PMC7164622; doi:10.1371/journal.pone.0230786)
Supplement: S2 Table — (DOCX) [file pone.0230786.s003.docx]

**Table S2 Primers used in this study**

| Name | Sequence5’-3’ | Usage |
| --- | --- | --- |
| Rv2498cf | ACTAGAATTCTGATGAACCTGCGTGCCGCCGG | Clone and expression |
| Rv2498cr | GCTGACTCTAGATCATTCGGAGGTGGCTTCCC | Clone and expression |
| E36A-Af  E36A-Ar  E36A-Bf  E36A-Br  E37A-Af  E37A-Ar  E37A-Bf  E37A-Br  R64A-Af  R64A-Ar  R64A-Bf  R64A-Br  E112A-Af  E112A-Ar  E112A-Bf  E112A-Br  D138A-Af  D138A-Ar  D138A-Bf  D138A-Br  R160A-Af  R160A-Ar  R160A-Bf  R160A-Br  CitEΔC50f  CitEΔC50r  B2518cUP-f  B2518cUP-r  B2518cDown-f  B2518cDown-r  Probe-f  Probe-r | ATGAACCTGCGTGCCGCCGGTCCGGGGT GGCCACGCCGTCGGCGAGGTCGAGAATC GATTCTCGACCTCGCCGACGGCGTGGCC TCATTCGGAGGTGGCTTCCCCGGCTCGCCG  ATGAACCTGCGTGCCGCCGGTCCGGGG  TCGGCCACGCCGGCCTCGAGGTCGAG  GGTGATTCTCGACCTCGAGGCCGGCGTGGC  TCATTCGGAGGTGGCTTCCCCGGCTCGCCG  ATGAACCTGCGTGCCGCCGGTCCGGGGT  CCGCCCGCATTGATGGCCACCACCGTGCGT ACGCACGGTGGTGGCCATCAATGCGGGCGG  TCATTCGGAGGTGGCTTCCCCGGCTCGCCG ATGAACCTGCGTGCCGCCGGTCCGG  GCGCGGTAGCCACCAGCGCGATGAC  GTCATCGCGCTGGTGGCTACCGCGC TCATTCGGAGGTGGCTTCCCCGGCTCGCCG  ATGAACCTGCGTGCCGCCGGT  GGTGGCGATCAAGGCTTCGGCACCCCACA  TGTGGGGTGCCGAAGCCTTGATCGCCACC  TCATTCGGAGGTGGCTTCCCC  ATGAACCTGCGTGCCGCCGGT  AGCGGACATGGGCGGCCACGTCACGATAG  CTATCGTGACGTGGCCGCCCATGTCCGCT  TCATTCGGAGGTGGCTTCCCC  ACTAGAATTCTGATGAACCTGCGTGCCGCCGG  GCTGACTCTAGATCAGGGTCGATAGGCCTTGC  GTGCTTAATTAATATGCCAACTTCTTGTCCAT  TGATACTAGTTCATTGCGCCTCCTTAATGG  TAGTGCTAGCGGGCGCAGATCCCCCGCGAC  AGTCGCTAGCCGGCAGCTTCGGCCATCACC  GAGTTCCAGATCGGCACCACAT  GGTCTCTGCATAAAGCGTGTCG | Clone and expression  Clone and expression  Clone and expression  Clone and expression  Clone and expression  Clone and expression  Clone and expression  Clone and expression  Clone and expression  Clone and expression  Clone and expression  Clone and expression  Clone and expression  Clone and expression  Clone and expression  Clone and expression  Clone and expression  Clone and expression  Clone and expression  Clone and expression  Clone and expression  Clone and expression  Clone and expression  Clone and expression  Clone and expression  Clone and expression  Knock out  Knock out  Knock out  Knock out  Southern blotting  Southern blotting |

Notes: Mutation sites designed are underlined.
