# Supplementary material for: Citrate lyase CitE in Mycobacterium tuberculosis contributes to mycobacterial survival under hypoxic conditions
Source: PLoS One. 2020 Apr 17;15(4):e0230786. doi: 10.1371/journal.pone.0230786 (PMC7164622; doi:10.1371/journal.pone.0230786)
Supplement: S3 Table — (DOCX) [file pone.0230786.s004.docx]

**Table S3 Primers for quantitative real-time PCR (qRT-PCR)**

| Name | Sequence 5’-3’ | Usage |
| --- | --- | --- |
| Rv2498cRTf | ACGTGGTGATTCTCGACCTC | qRT-PCR |
| Rv2498cRTr | AGCATCACCGTGGTATAGGC | qRT-PCR |
| Rv2454cRTf | GATCTGTCGGTATGGGTGGT | qRT-PCR |
| Rv2454cRTr | CGGCGAATACTGTCCTTTGG | qRT-PCR |
| Rv2455cRTf | CACGAACTGTCCAAGCACAA | qRT-PCR |
| Rv2455cRTr | CACGTCTATGACCAGCAACG | qRT-PCR |
| Rv0757cRTf | GGCTTTGAAGTCTACACCGC | qRT-PCR |
| Rv0757cRTr | ACATAGTCGTCACCACCCAG | qRT-PCR |
| Rv2031cRTf | TGAAAGAGGGGCGCTACGAG | qRT-PCR |
| Rv2031cRTr | GTCCTCGTCAGCACCTACCG | qRT-PCR |
| SigAf | TCGCGCCTACCTCAAACAG | qRT-PCR |
| SigAr | CGTACAGGCCAGCCTCGAT | qRT-PCR |
