# Supplementary figures and images for: Citrate lyase CitE in Mycobacterium tuberculosis contributes to mycobacterial survival under hypoxic conditions
Source: PLoS One. 2020 Apr 17;15(4):e0230786. doi: 10.1371/journal.pone.0230786 (PMC7164622; doi:10.1371/journal.pone.0230786)

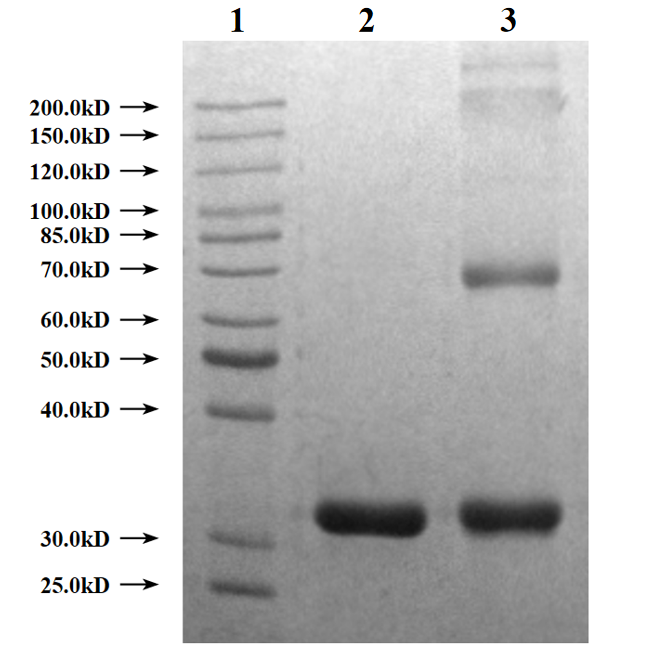

Supplement: S1 Fig — Oligomeric status of the CitE protein was detected by Chemical Cross-Linking assay according to previous reports. The 20 μl reaction mixes, including 10 mM CitE protein, 4 μM Disuccinimidyl suberate (DSS) and cross-linking buffer (100 mM NaH2PO4 pH 8.0 and 150 mM NaCl), were incubated at room temperature. Half an hour later, the reaction was stopped by incubating with standard SDS-PAGE sample buffer at 100 °C for 5 min, and then analyzed by SDS-PAGE. (TIF) [file pone.0230786.s005.tif]

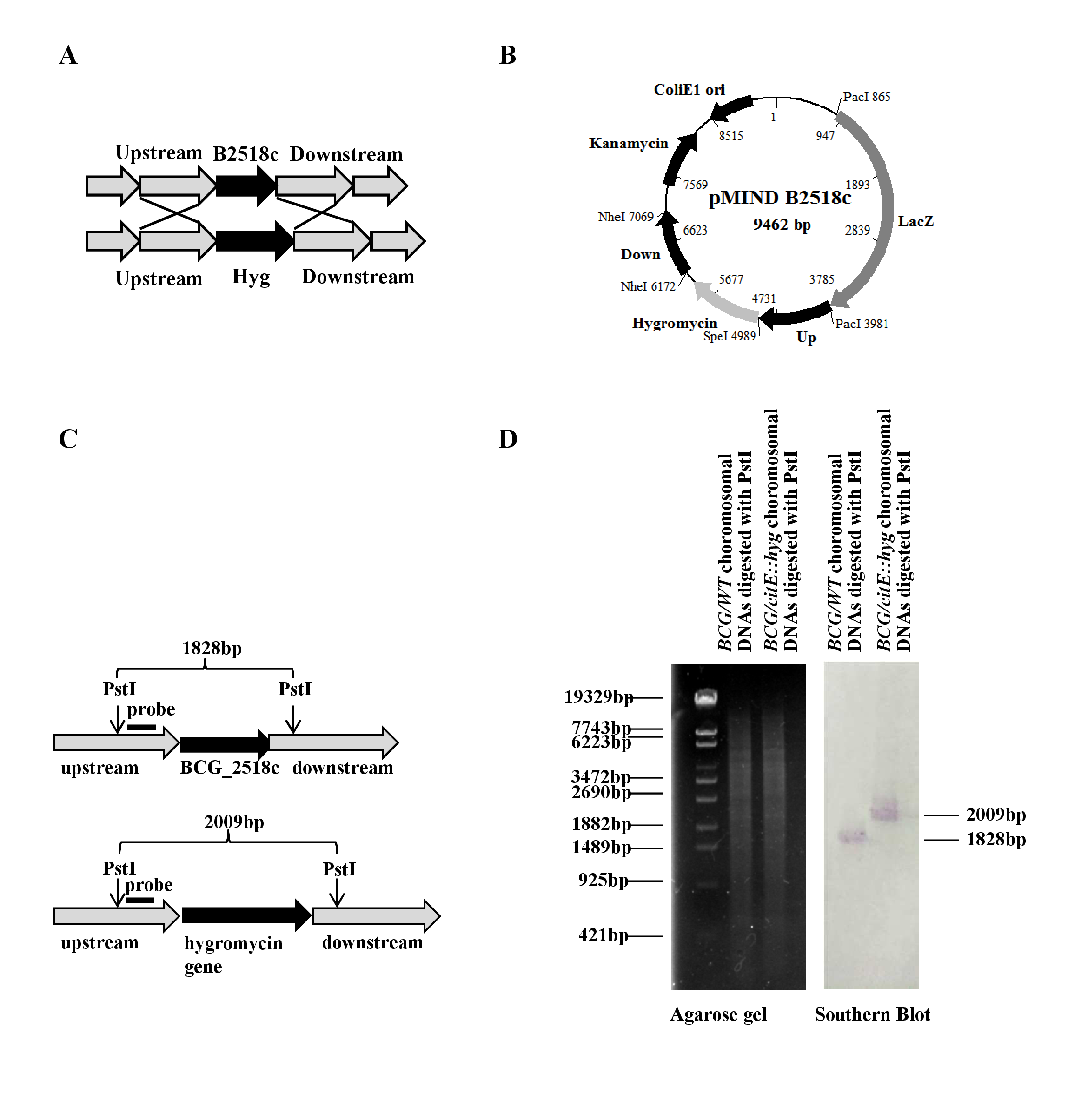

Supplement: S2 Fig — (A) The double-crossover recombinant strategy for M. bovis citE gene knockout. Upstream, 1 kb genomic DNA of citE gene upstream; Downstream, 1 kb genomic DNA of citE gene downstream; Hyg, hygromycin resistance gene. (B) A map of the recombinant vector constructed for citE knockout. Upstream DNA fragment was inserted between Pac I and Spe I sites of the vector; downstream was placed at Nhe I site of the vector; LacZ gene was put into Pac I site of the vector. (C) Schematic of genomic DNA from wild-type and citE knockout strains digested with the restriction enzyme Pst I. The probe is indicated with a black bar. (D) Southern blot assays. Southern blotting was performed to detect genomic DNA containing citE gene or hygromycin gene. A 300-bp probe corresponding to the digested fragment was obtained by PCR and labeled with digoxigenin dUTP (Roche, Mannheim Germany). The samples were hybridized with the external probe, and they showed changes in the size before and after recombination. (TIF) [file pone.0230786.s006.tif]

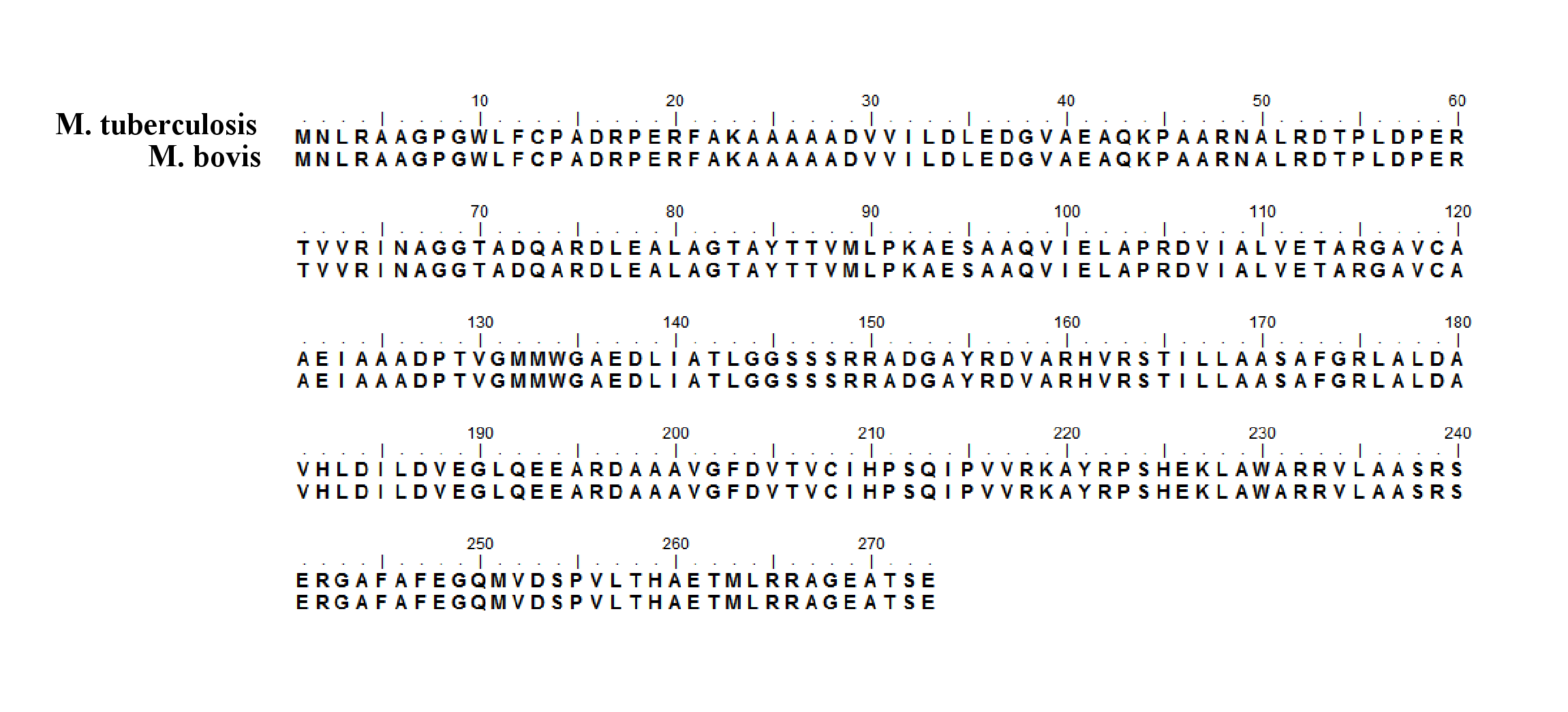

Supplement: S3 Fig — The sequence alignment of amino acids shows the homology of the citE protein in TB to BCG are 100% identity. (TIF) [file pone.0230786.s007.tif]

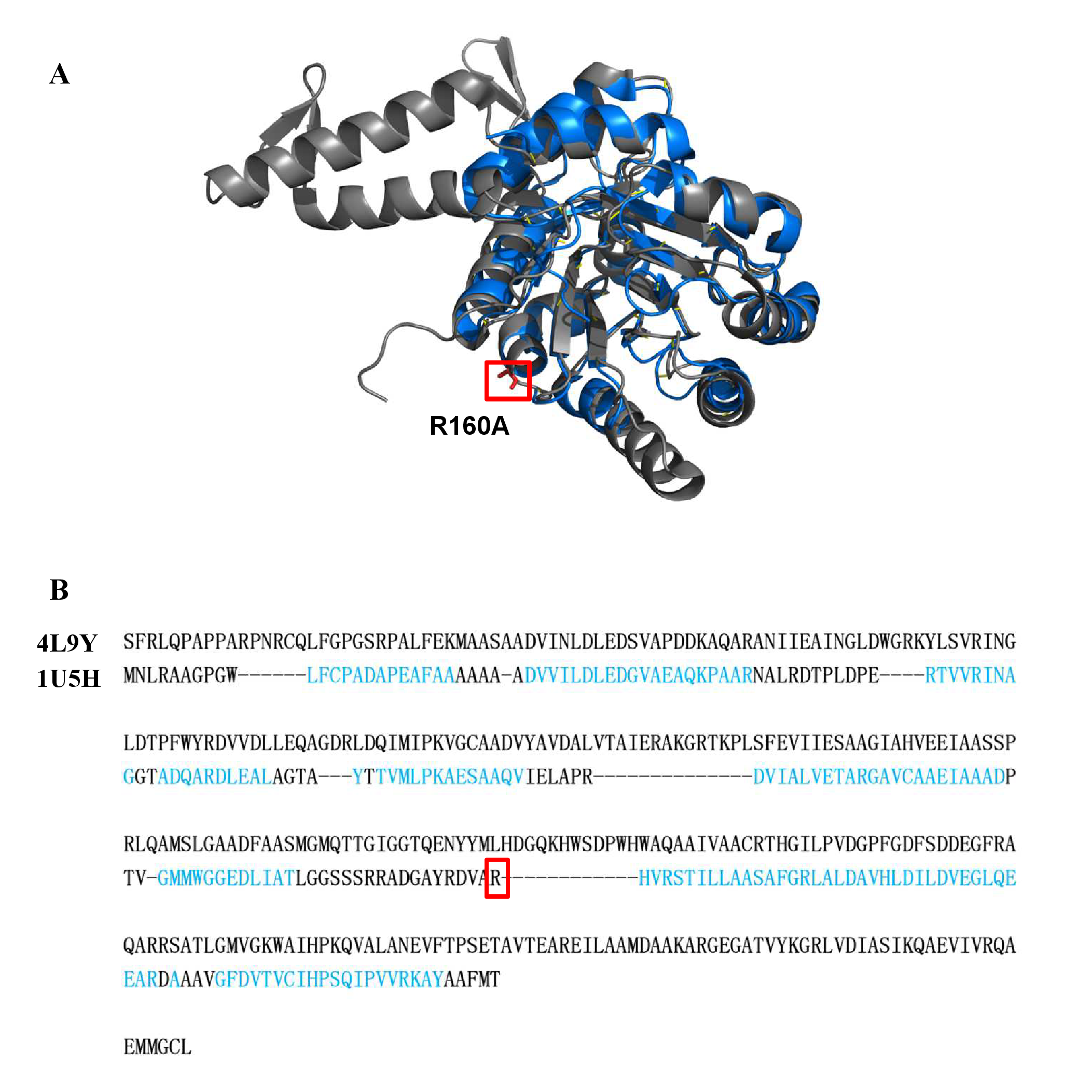

Supplement: S4 Fig — (A) Overlay of R. sphaeroides malyl-CoA lyases and MtbCitE. MCL (PDB 4L9Y) is colored grey and CitE (PDB 1U5H) is colored in blue. R160 of MtbCitE is showed in red sticks. (B) Comparison of MCL and CitE in sequence. The residues overlaid with MCL in structure colored blue in CitE sequence. R160 of MtbCitE is indicated by red box. (TIF) [file pone.0230786.s008.tif]

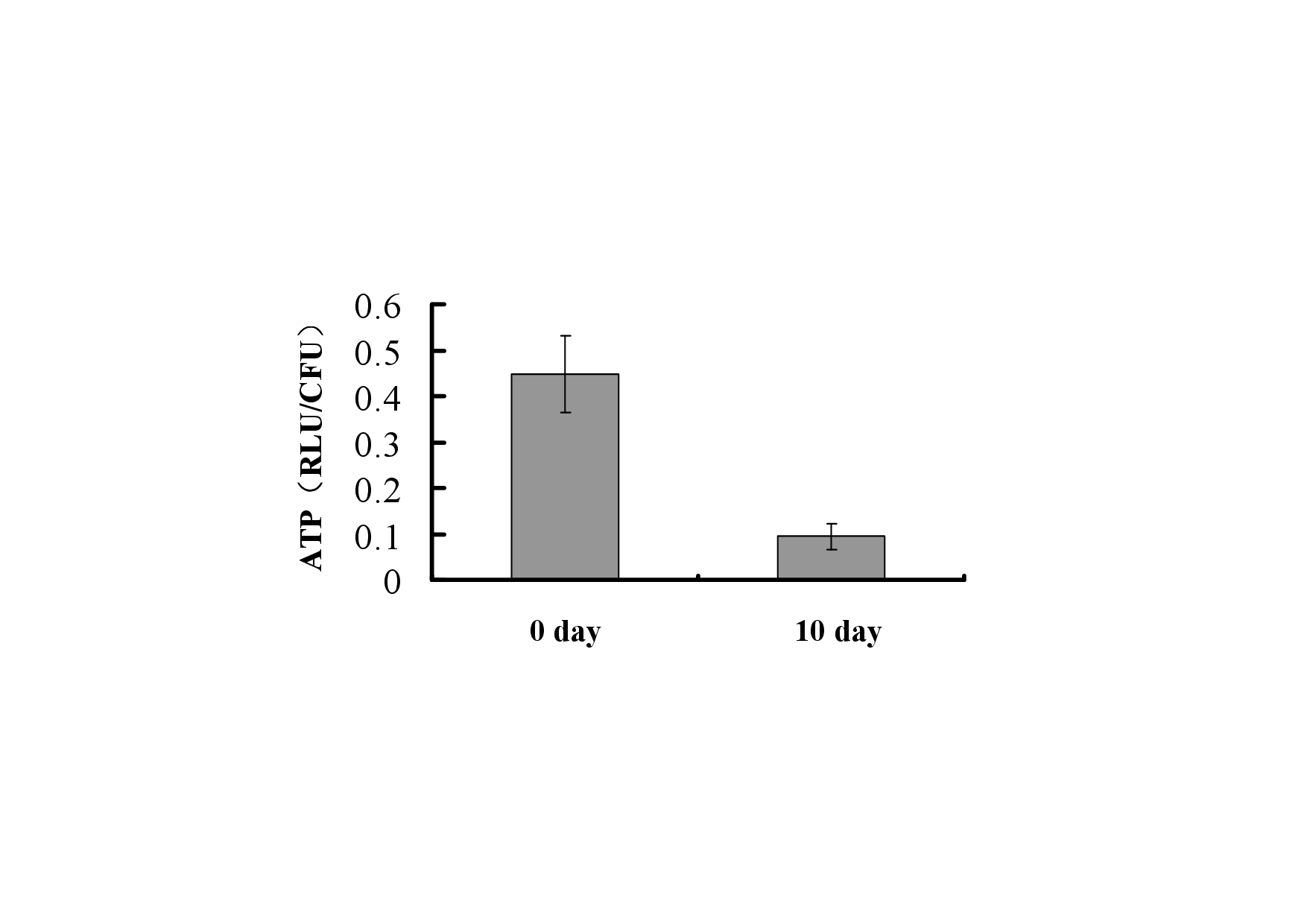

Supplement: S5 Fig — The M. bovis BCG was cultured and collected according to the Materials and Methods. (TIF) [file pone.0230786.s009.tif]
